# University of Michigan

**Published:** 1883-04

**Authors:** 


### University of Michigan.

THE eighth annual commencement of the Dental Department of the Uuniversity of Michigan was held on Wednesday, March 28, at 2 o'clock P.M., in University Hall. A very interesting address was delivered by Dr. G. W. Thomas, of Detroit, in the presence of a large audience.

The degree of doctor of Dental Surgery was conferred upon the following persons, viz. :

| NAME. | RESIDENCE. |
|---|---|
| Frederick Hornby Berry,...................... | Ann Arbor. |
| Charles Blair Blackmarr,...................... | Jackson. |
| Wilbur Buzzell,...................................... | Flint. |
| Maximilian E. Chapalay,..................... | Bucharest Roumania. |
| Bernard Henry Conlin, ....................... | Columbus, Wis. |
| William Walter Curtis, ....................... | Chicago, Ill. |
| Walter Irving Dadmun,....................... | Milwaukee, Wis. |
| Marshall Bidwell Dennis,..................... | New Market, Ont. |
| William Otis De Puy, .......................... | Ann Arbor. |
| George Lewis Fox,................................ | Adams Centre, N. Y. |
| John William Gale, .............................. | Lodi, N. Y. |
| Charles A. Gallagher, .......................... | South Bend, Ind. |
| Arthur St. Clair Graham, .................. | Colfax, Ohio. |
| Will Harmon Hall,............................... | Martin's Ferry, Ohio. |
| Stanley Read Holden,.......................... | Watkins, N. Y. |
| Frank Alexander McAuley, ............... | Muskegon. |
| Charles Cornelious Newcastle,............. | Grand Rapids. |
| William Franklin Overholser, ............. | Logansport, Ind. |
| Byron Smith Palmer,........................... | Coldwater. |
| Lyman Trumbull Phillips,.................... | Nashville, Ill. |
| Perley Andrews Powers,...................... | Hollis, N. H. |
| Ozora Pierson Sutherland, .................. | Monroe, Wis. |
| John Brinkerhoof Van Fossen,............ | Ypsilanti. |

Matriculates during the term sixty-nine.

